# Use of Fibrin Glue as a Surgical Adjunct in Bone Grafting of Fracture Non-unions

**DOI:** 10.5704/MOJ.2407.007

**Published:** 2024-07

**Authors:** R Kunnasegaran, JW Ng, EBK Kwek

**Affiliations:** 1 Department of Orthopaedic Surgery, Tan Tock Seng Hospital, Singapore; 2 Department of Orthopaedic Surgery, Woodlands Health, Singapore

**Keywords:** fractures, ununited, fibrin tissue adhesive, bone transplantation, fracture healing

## Abstract

**Introduction:**

Non-union of long bones is a common challenge in the treatment of fractures. Bone grafting is commonly used to treat atrophic non-union, but mechanical displacement of the graft may occur, resulting in delay or failure of treatment. Fibrin glue has demonstrated positive results in management of bone defects in neurosurgery and oromaxillary facial surgery, however, there has yet to be any study on its use in long bone fractures.

**Materials and Methods:**

We conducted a prospective randomised controlled trial at a single tertiary centre involving adult patients with long bone fractures that had undergone non-union and requiring bone grafting only. Autologous iliac crest bone graft was applied to the debrided non-union site, with additional fibrin glue applied for the intervention arm. Patients were followed-up with serial radiographs until clinical and radiographical union.

**Results:**

Ten patients (3 male, 7 female), of mean age 41.7 (19 – 63) were recruited over five years, with one drop out. Eight out of nine fractures united after treatment. One patient underwent hypertrophic non-union requiring re-fixation and bone grafting. There was no difference in the time to union for patients in the fibrin glue group (19.5 weeks) versus the control group (18.75 weeks) (p=0.86). There were no complications sustained from usage of fibrin glue.

**Conclusion:**

Fibrin glue appears to be a safe adjunct for treatment of non-union of long bone fractures across varying fracture sites by holding the bone graft in place despite not demonstrating a faster time to union.

## Introduction

Orthopaedic surgeons commonly face the common yet challenging dilemma of fracture non-union. It is characterised by cessation of normal biological healing of the bone with additional interventions required before further healing can occur. It has been defined as a fracture not radiologically healed by six months (United States Food and Drug administration) or as a fracture that has not displayed progression of healing at three months^1^. Atrophic non-union occurs in the absence of adequate vascular supply, with resultant lack of callus formation^2^.

Autologous bone graft with its osteogenic, osteoinductive and osteoconductive properties has classically been the gold standard in treatment of atrophic non-union^3^. One draw-back to bone grafting is the concern of mechanical displacement of the harvested bone graft at the recipient site.

TISSEEL glue [Baxter, Deerfield, Illinois, United States of America] is a fibrin sealant derived from pooled human plasma. It comprises of human derived fibrinogen with synthetic fibrinolysis inhibitor Aprotinin, that when mixed with the thrombin component results in fibrinogen being cleaved by the protease thrombin with formation of a fibrin clot. This mimics the final stage of the blood coagulation cascade. It has been utilised for hemostasis, reinforcement of vascular and bowel anastomosis and wound management^4^. Despite this, there have been no studies on effects of fibrin sealant with regards to treatment of long bone non-union.

For our study, our primary objective was to assess whether the addition of fibrin sealant to bone graft would result in a shorter time to union than bone graft alone in the treatment of atrophic non-union of long bone fractures. Our secondary objective was to assess for the safety profile of fibrin sealant in treatment of non-union.

## Materials and Methods

This prospective double-blind randomised controlled pilot trial was conducted at a single tertiary hospital in Singapore after obtaining ethics board approval. This study was designed according to CONSORT guidelines and was funded by a grant from AO Trauma Asia Pacific (Project number AOTAP 13-09).

Patients between the age of 21 and 70 who sustained an open or closed fracture of any long bone that was treated conservatively or surgically which subsequently underwent non-union that was deemed to require bone grafting only were recruited. Recruitment was over a 5-year period from February 2015 to April 2020. Femur, tibia, humerus, radius and ulna fractures with subsequent non-union were included for evaluation.

Patient demographics, medical history, initial fracture treatment, subsequent non-union treatment and operative and follow-up data was collected.

A diagnosis of non-union was made based on findings of local tenderness and fracture site motion on clinical examination and radiographical evidence of absence of bone bridging on three of four cortices on orthogonal radiographs at six months after injury or lack of progression of healing on serial radiographs over a three-month period^5^.

Patients with ASA grades of V and VI, with a history of infected non unions, pathological fractures, patient receiving immunosuppressive agents or corticosteroids, patients deemed unfit for surgery, pregnant patients, patients with non-union secondary to mechanical instability and patients who failed previous bone grafting procedures were excluded from this study.

Radiographs were reviewed by an orthopaedic trauma consultant and patients were selected based on the abovementioned inclusion criteria. Eligible patients were contacted by the study team and offered recruitment. Written informed consent was obtained from all participants. Randomisation was performed using sealed, opaque envelopes. The sequence was generated by a random number generator. Patients were allocated to either bone graft alone or bone graft with fibrin sealant in a one: one ratio and were kept blinded to the intervention they underwent. Patient outcome measures and radiographs were anonymised and were assessed by the trauma consultant not involved in their care.

Surgery involved debridement of the non-union site of fibrous tissue until healthy bone was exposed. Tissue was obtained and sent for cultures. The pre-existing fixation was assessed for stability and if deemed unstable and requiring revision of the fixation, the patient would be excluded from the study.

Morselised cancellous bone autograft was obtained from the ipsilateral anterior iliac crest and impacted into the site of non-union. In the fibrin glue treatment arm, TISSEEL glue was injected into and applied over the bone graft as a sealant. The exact amount of TISSEEL glue applied was dependent on the size of the defect and amount of bone graft used, up to a maximum of 4ml. This was followed by layered closure in both groups.

Patients were followed-up post-operatively in the outpatient orthopaedics clinic at two weeks and subsequently every four weeklies until evidence of fracture healing was seen. Patients were followed-up for a minimum of 12 months post-injury. At each visit, pain, ambulation status, complications from the surgery as well as clinical examination was performed, assessing for local tenderness, wound healing, sensation of instability and ability to ambulate (if applicable). Radiographs were obtained at each visit. Fracture healing was established when there was lack of pain during weight bearing and bridging of three out of four cortices in the obtained radiographs^5^.

IBM SPSS Statistics Version 25.0 was used for data analysis. Non-parametric, continuous variables were tested with Mann Whitney test while categorical variables were tested with Fisher's exact test for any significant statistical differences between the two groups. Parametric, normally distributed variables were tested with two tailed Student’s t-test. A p-value of <0.05 was regarded as statistically significant.

## Results

Ten patients (7 female, 3 male), of mean age 41.7 (19 – 63) were recruited. The patients were split into 2 groups, with 5 patients in each arm. Both groups were similar in terms of demographics (Table I). Patients with non-union of the humerus, radius, ulna, femur and tibia were recruited (Table II). The study was terminated in December 2020 due to a paucity of suitable patients.

**Table I:** Study population demographics.

Variable	Bone Graft (n = 5)	Bone Graft with Fibrin glue (n = 5)	P-Value
Age (range, mean)	30 – 63 (45.2)	19 – 57 (38.2)	0.4625
BMI (range, mean)	19.2 – 30.6 (26.05)	23.1 – 29.5	
(26.875)	0.7808		
Gender	2 Male 3 Female	1 Male 4 Female	1
Smoking (n,%)	0	2 (40)	0.4286
Open fracture (n,%)	1 (20)	3 (60)	0.5238
Time to revision surgery (weeks) (Range, mean)	9 – 25 (12.4)	9 – 15 (9)	0.4095

**Table II:** Site of non-union.

Site of non-union	Bone Graft (n = 5)	Bone Graft with Fibrin glue (n = 5)
Humerus	1	0
Radius/Ulna	1	1
Femur	2	3
Tibia	1	1

**Fig. 1:**
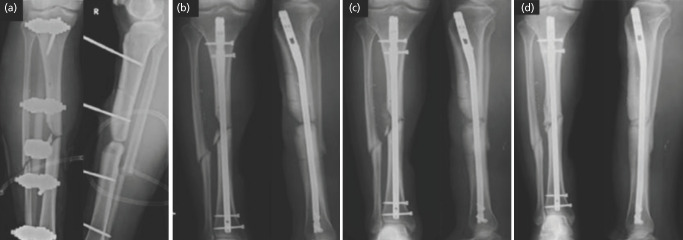
(a) Clinical radiographs of a patient with a Gustilo 3C open tibia fracture that underwent intra-medullary nailing and a pedicled hemisoleus flap. (b) This was complicated by non-union and (c) underwent bone grafting with fibrin glue (d) with subsequent union at 28 weeks.

One patient in the bone graft only group was withdrawn from the study due to a subsequent diagnosis of metastatic cancer. Eight out of nine patients eventually united (Fig. 1). A 46-year-old female with a Gustilo one open left femur shaft fracture in the bone graft and fibrin glue group developed hypertrophic non-union. She subsequently underwent revision nailing with a larger nail and iliac crest bone grafting 10 months after her initial bone grafting. She subsequently demonstrated complete healing at 28 months post-op.

There was no statistically significant difference between both groups for gender, age, BMI, smoking, presence of open fracture or time to revision. There were no patients with end stage renal failure, peripheral vascular disease, osteoporosis or other rheumatological conditions. One patient in the fibrin glue and bone graft group had type 2 diabetes mellitus and 1 patient in the bone graft only group had Sjogren’s syndrome but was not on corticosteroids or immunosuppressants.

The mean time taken for patients in the fibrin glue group to undergo healing was 19.5 weeks as compared to the 18.75 weeks for patients in the bone graft only group (p=0.86). There were no complications arising directly from the usage of the fibrin glue.

## Discussion

Fibrin sealants have been used extensively in surgeries with few reported adverse effects^4-6^. It has been used for haemostasis in surgeries where significant bleeding is expected for example after joint or bone surgery^7^ or cardiothoracic surgery^6,8^. Other indications include the coating and sealing of vascular prostheses, reinforcement of bowel anastomoses^4^ and as adhesive for skin graft placement^9^. The above mentioned are only a few of the many different circumstances during which fibrin sealants have shown to be beneficial.

In the treatment of non-union, the diamond concept of bone fracture healing talks about the interaction between potent osteogenic cell populations, an osteoconductive matrix scaffold, osteoinductive stimulus and mechanical stability to create the most optimal biological environment for osteogenesis^10,11^.

We have demonstrated that fibrin glue appears to be a safe adjunct for treatment of non-union in long bone fractures. This was consistent across different fracture sites. While union rates in both groups were similar, this may arise from limitations in our sample size not allowing for pick-up of differences in the rate of union.

From a biological standpoint – a critical analysis of the pre-existing literature reveals that fibrin glue alone does not have any osteoinductive properties in canine^12^, rabbit^13^ and rodent bone models^14^. However, when fibrin glue is combined with bone graft in animal models^15-17^ or in oromaxillary facial surgery^18,19^ and neurosurgery^20,21^, it has consistently demonstrated positive results as compared to bone graft alone. In the surgical treatment of alveolar clefts, Segura-Castillo *et al*^18^ described reduced bone resorption with improved graft integration and quality when fibrin glue was combined with the bone graft. Tayapongsak’s^19^ series of 33 cases of mandibular reconstruction with particulate cancellous bone and fibrin glue showed earlier bony incorporation and remodelling detected radiographically at the fourth week as compared to eight weeks in bone grafts alone.

We hence believe that fibrin glue plays a key role in the diamond concept by augmenting the effectiveness of cancellous bone grafting by providing early mechanical stability for the cancellous bone graft by clot formation at the recipient site, providing a good scaffold containing numerous growth factors for proliferation of osteo-induced bone marrow stromal cells as evidenced in animal studies^22,23^. This was observed intra-operatively where there was subjectively less displacement of the bone graft when the fibrin glue was applied, and a clot formed. Objective assessment of evidence of displacement of the bone graft on radiographs was difficult but may be possible with higher resolution CT scans.

The safety of fibrin glue is evidenced by our patients who underwent bone grafting with fibrin glue application not having a longer time to union as compared to bone graft alone or any complications arising from fibrin glue application. We attribute this to the excellent biocompatibility of fibrin glue resulting in no direct effect on the pre-existing mesenchymal stem cells, osteocytes, osteoblasts and bone morphogenic proteins. Its angiogenic properties may also assist the neovascularisation process in the early stages of graft incorporation^24,25^. None of the known side effects of fibrin glue such as infection, hypersensitivity reactions or embolism^4^ were encountered during this study.

Even in the case of the patient who underwent revision nailing due to hypertrophic non-union after iliac crest bone grafting augmented with fibrin glue, there was evidence that suggested the local biological deficiencies that caused the atrophic non-union was addressed on the index bone grafting surgery. However, it is important to revisit the diamond concept and adequately address the mechanical stability as a key component in successful treatment of non-unions.

While our study was terminated early due to a lack of suitable patients, we found that most non-unions in our institution underwent revision of the pre-existing fixation in addition to bone grafting. It may be of interest to look at the role of fibrin glue as an adjunct for treatment of those injuries as well. It would be also interesting to quantify the amount of bone graft retention, possibly with post-operative computed tomography scans in future studies.

Limitations for this study includes a small sample size, heterogeneity of fracture patterns, varied location of non-union and varying defect sizes. Further comparative studies are needed to further evaluate its relative efficacy when compared to traditional bone grafting methods and its usage together with other adjuncts in the treatment of non-union of long bone fractures in orthopaedics.

## Conclusion

In conclusion, while the use of fibrin glue did not appear to result in a faster time to union, we found that it did not interfere with the bone healing process and was a safe and useful adjunct for sealing the bone graft in place.
